# Short-term Reproducibility of Pulmonary Nodule and Mass Detection in Chest Radiographs: Comparison among Radiologists and Four Different Computer-Aided Detections with Convolutional Neural Net

**DOI:** 10.1038/s41598-019-55373-7

**Published:** 2019-12-10

**Authors:** Young-Gon Kim, Yongwon Cho, Chen-Jiang Wu, Sejin Park, Kyu-Hwan Jung, Joon Beom Seo, Hyun Joo Lee, Hye Jeon Hwang, Sang Min Lee, Namkug Kim

**Affiliations:** 1 Department of Biomedical Engineering, Asan Institute of Life Science, University of Ulsan College of Medicine, Asan Medical Center, Seoul, South Korea; 2 Department of Radiology, The First Affiliated Hospital of Nanjing Medical University, Nanjing, China; 3 VUNO Inc., Seoul, South Korea; 4 Department of Radiology, University of Ulsan College of Medicine, Asan Medical Center, Seoul, South Korea; 5 Department of Convergence Medicine, University of Ulsan College of Medicine, Asan Medical Center, Seoul, South Korea

**Keywords:** Electrical and electronic engineering, Mechanical engineering

## Abstract

To investigate the reproducibility of computer-aided detection (CAD) for detection of pulmonary nodules and masses for consecutive chest radiographies (CXRs) of the same patient within a short-term period. A total of 944 CXRs (Chest PA) with nodules and masses, recorded between January 2010 and November 2016 at the Asan Medical Center, were obtained. In all, 1092 regions of interest for the nodules and mass were delineated using an in-house software. All CXRs were randomly split into 6:2:2 sets for training, development, and validation. Furthermore, paired follow-up CXRs (n = 121) acquired within one week in the validation set, in which expert thoracic radiologists confirmed no changes, were used to evaluate the reproducibility of CAD by two radiologists (R1 and R2). The reproducibility comparison of four different convolutional neural net algorithms and two chest radiologists (with 13- and 14-years’ experience) was conducted. Model performances were evaluated by figure-of-merit (FOM) analysis of the jackknife free-response receiver operating curve and reproducibility rates were evaluated in terms of percent positive agreement (PPA) and Chamberlain’s percent positive agreement (CPPA). Reproducibility analysis of the four CADs and R1 and R2 showed variations in the PPA and CPPA. Model performance of YOLO (You Only Look Once) v2 based eDenseYOLO showed a higher FOM (0.89; 0.85–0.93) than RetinaNet (0.89; 0.85–0.93) and atrous spatial pyramid pooling U-Net (0.85; 0.80–0.89). eDenseYOLO showed higher PPAs (97.87%) and CPPAs (95.80%) than Mask R-CNN, RetinaNet, ASSP U-Net, R1, and R2 (PPA: 96.52%, 94.23%, 95.04%, 96.55%, and 94.98%; CPPA: 93.18%, 89.09%, 90.57%, 93.33%, and 90.43%). There were moderate variations in the reproducibility of CAD with different algorithms, which likely indicates that measurement of reproducibility is necessary for evaluating CAD performance in actual clinical environments.

## Introduction

In general clinical practice, chest radiography (CXR) is usually the first choice of imaging for patients with nonspecific symptoms for thoracic conditions. As CXR is easily available and inexpensive, it became a part of screening tool to detect a disease in its earliest stages. However, there are several practical limitations for radiologists in assessing the results while maintaining a high quality of diagnosis; in fact, frequently missed diagnoses even by experienced radiologists were detected retrospectively^1–3^.

CAD has been introduced to help radiologists and showed added value in the detection of pulmonary nodules in CXR^4–6^. Hoop, *et al*.^7^ demonstrated that the sensitivity of CAD is comparable to that of expert radiologists in identifying lung cancer with low-dose computed tomography (CT) screening. However, the sensitivity of stand-alone CAD in follow-up (F/U) CXR was found to be 71% with 1.3 false-positive findings per image^8^. Although CAD performance has improved significantly, it still requires better sensitivity and specificity to be integrated into routine clinical practice.

Several different types of CAD systems have been recently implemented as part of the picture archiving and communication system (PACS) technology^9–14^. In CXR, the chest CAD package might include automated detection of lung nodules, interstitial opacities, cardiomegaly, vertebral fractures, and interval changes. Moreover, deep learning with convolutional neural net (CNN) algorithms have been successfully adapted in computer vision technology and CAD in CXRs for the detection and classification of multiple lesions. Lakhani *et al*.^11^ showed that deep-learning techniques can accurately classify tuberculosis in CXR with an area under the curve (AUC) of 0.99, which is higher than that described in a previous study (AUC of 0.87–0.90), with support vector machines^10^. Similarly, Islam *et al*.^9^ studied CXR-based diagnosis of pulmonary abnormalities and demonstrated a high performance in the ensemble deep-learning model.

To introduce this novel technique in actual clinical practice, one of the most important requirements is reproducibility as there are several variable parameters, such as breathing, posture, position, and device settings, that should be taken into account. However, there is not much information available on CAD reproducibility. Kumar *et al*.^15^ evaluated the reliability and validity of CXR between the best physician and best radiologist in the diagnosis of pulmonary tuberculosis. To the best of our knowledge, reproducibility of CAD based on CNN has not yet been intensively evaluated. In this study, we propose that reproducibility is an important indicator of CAD performance for clinical purposes.

Thus, we investigated the reproducibility of CAD with four different convolutional neural net algorithms such as Mask R-CNN^16^, RetinaNet^17^, YOLO (You Look Only Once) v2^18^-based eDenseYOLO, and atrous spatial pyramid pooling^19^ (ASPP) -based U-Net^20^ and two chest radiologists (with 13- and 14-years’ experience) for chest radiography (CXR) of the same patient with nodules and masses within a short-term period.

## Materials and Methods

### Subjects

The institutional review board for human investigation at the Asan Medical Center (AMC) approved our study protocol with removal of all patient identifiers from the images. The need for informed consent was waived due to the retrospective nature of this study.

**Figure 1:**
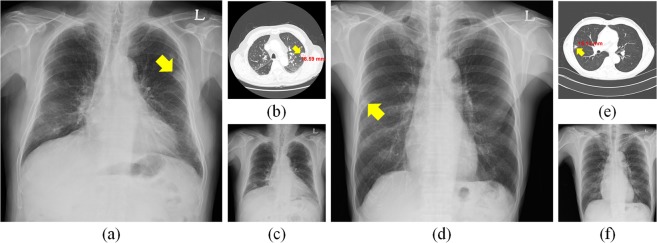
Initial and follow-up (F/U) CXRs and CT images with a nodule. (**a**) Initial CXR in a 65-year-old male patient with 18.59 cm metastatic renal cell carcinoma (arrowhead) in the left upper lobe and (**b**) CT examination of patient (**a**). (**c**) F/U CXR of (**a**). (**d**) Initial CXR of an 81-year-old male patient with 15.13 cm metastatic adenocarcinoma (arrowhead) in the right middle lobe and (**e**) CT examination corresponding to (**d**). (**f**) F/U CXR of (**d**).

A total of 944 CXRs (Chest PA) with pulmonary nodules or masses captured between January 2010 and November 2016 at the AMC were obtained. Later, a total of 1092 regions of interest (ROIs) of the nodules or masses in initial CXRs were delineated by expert thoracic radiologists by consensus using an in-house software on the nearest corresponding CT images as the ground truth. The CXRs were randomly split into 6:2:2 sets for training, development, and validation, respectively. The average time interval between initial and F/U CXRs was (4.00 ± 3.69) days; the average interval between initial CXRs and CT scans captured at AMC was (5.92 ± 13.52) days while that in the case of CTs captured in other hospitals was (13.42 ± 8.53) days. To measure reproducibility, only 121 paired CXRs in the validation set were enrolled depending on the availability of F/U CXRs; these were recorded within one week and no disease change was confirmed by expert thoracic radiologists (Fig. 1). Detailed demographics were listed in Table 1.

**Table 1:** Demographics corresponding to training and validation sets.

Characteristic		Training and development set (N = 822 single CXRs)	Validation set (N = 121 paired CXRs)
Age (per patient)		61.24 ± 10.74	60.74 ± 9.12
Male		632 (76.96%)	90 (74.38%)
Multiple lesions	One lesion	740	108
	Multiple lesions (≥2)	82	13
	Total	822	121
Size (mm)	≤10 mm	14 (1.49%)	2 (1.39%)
	10–20 mm	117 (12.45%)	21 (14.58%)
	20–30 mm	231 (24.57%)	23 (15.97%)
	≥30 mm	578 (61.49%)	98 (68.06%)
	Total	940	144
Location	Right upper	278 (29.57%)	45 (31.25%)
	Right middle	80 (8.51%)	12 (8.33%)
	Right lower	144 (15.32%)	24 (16.67%)
	Left upper	244 (25.96%)	37 (25.69%)
	Left lower	194 (20.64%)	26 (18.06%)
	Total	940	144

### Methods

To evaluate the reproducibility of CAD for nodules and masses, four different algorithms were trained and two board-certificated chest radiologists (R1 and R2 with 14- and 13-years’ experience, respectively) participated in this study.

We used different architectures based on CNN, viz. one-stage learning-based RetinaNet and modified eDenseYOLO, two-stage learning-based Mask R-CNN, and ASPP based U-Net. In this study, RetinaNet and Mask R-CNN were trained without architecture modification, while eDenseYOLO and ASPP U-Net were trained with modification from their original architecture to improve CAD performance. Simple augmentation methods, such as pixel windowing, histogram matching, rotation, blur, brightness, contrast, inversion, Gaussian noise, sharpness, shift, and zoom, were used when training Mask R-CNN, RetinaNet, and eDenseYOLO. For training ASPP U-Net, random crop, orientation, brightness adjustment, and Gaussian noise and Poisson noise were used as the augmentation methods. Detailed hyper-parameters were summarized in Table 2. Five different cut-off thresholds for reproducibility were determined empirically as the number of average false positives (0.1, 0.2, 0.3, 0.4, and 0.5) in the free-response receiver operating characteristic (FROC) curve for analyzing the reproducibility of the validation set. A hit-criterion was defined as intersection over union between labeled box and predicted box is over 0.5.

**Table 2:** Parameters used for training four different CNN-based algorithms for CAD.

Algorithm	Mask R-CNN	RetinaNet	eDenseYOLO	ASPP U-Net
Stages	Two stages	Single stage	Single stage	Single stage
Backbone	Resnet101	ResNet101	DensNet201	ResNet50
Optimizer	SGD	SGD	Adam	Adam
Learning rate	1e-6	1e-6	1e-3	1e-3
Weight decay	0.0	0.0	0.0005	–
Momentum	0.9	0.9	0.9	0.9, 0.999

### Description of the four algorithms

The Mask R-CNN algorithm is divided into two steps; the first extracts candidate regions as a region proposal network and the second classifies or segments them. This algorithm is designed not only to find object boundaries but also segment objects. Mask R-CNN with feature pyramid network (FPN) was used to train and infer nodules, which is more robust to various sizes of nodules than those with a single scale feature map.

RetinaNet is a one-stage detector and is simple and fast to train as the detection model. It is an FPN with cross-entropy loss replaced by focal loss and infers objects of different sizes at different scales in the feature map. The alpha and gamma values for focal loss were set at 0.25 and 2.0, respectively. Resnet50 was used as the backbone network and the three final layers were used for FPN.

**Figure 2:**
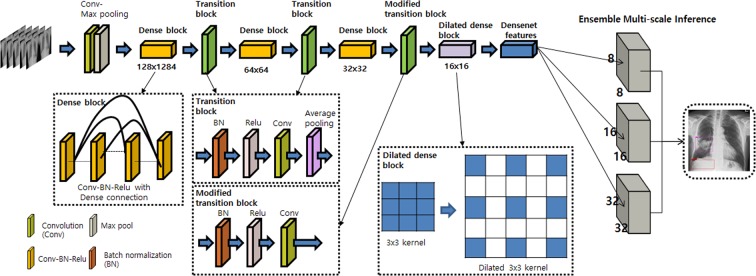
Architecture of eDenseYOLO with a backend network of DenseNet201. The output layers of eDenseYOLO, i.e., YOLO v2 with DenseNet201, were modified for improved robustness with respect to disease-pattern size. If the input is 256 × 256, the feature map for the last layer is 8 × 8, 16 × 16, or 32 × 32 with a skip connection.

We used the eDenseYOLO system, modified from its original YOLO architecture. The output layers of YOLO v2 with DenseNet201 as eDenseYOLO, modified to be robust at different nodule/mass sizes, are shown in Fig. 2. For example, if the input is 256 × 256, the feature map of the output layer takes various resolution forms, such as 8 × 8, 16 × 16, or 32 × 32. The output layer is concatenated to fuse information together. The output layer is modified to exploit context information from regions with different resolutions (ensemble), including pooled features for each feature map with a foveal structure. It is effective to train and predict abnormalities (objects) of different scales in chest radiographs. This network predicts class confidence scores and locations of bounding boxes to detect multiple lesions in CXRs with individual layers.

The ASPP U-Net, a modified version of the U-net architecture, was used to segment nodules and masses. The core of U-net architecture consists of an encoder-decoder scheme and a lateral skip connection. The encoder is followed by the ASPP layer to detect multi-scaled objects. ASPP uses atrous (dilated) convolutions of different rates to classify regions of an arbitrary scale. Atrous convolutions are special convolutions with a factor that expands the field of view. It expands (dilates) the convolution filter according to the dilation rate and fills the empty spaces with zeros, thereby creating a sparse filter. Using multiple parallel atrous convolutional layers with different sampling rates, we can aggregate a multi-scaled object detector into one model. To generate the bounding box and the corresponding confidence of a detected nodule, we performed connected component labeling to softmax the output map of the segmentation network with a given threshold (0.05). For every bounding box, the confidence was calculated by averaging the softmax value of each pixel inside the bounding box.

### Description of the two participating chest radiologists

Two expert thoracic radiologists (R1 and R2 with 14 and 13 years’ experience, respectively, in chest radiology) were recruited to verify the results of human assessment. All patient information, except for the CXRs, were blinded. Signs of possible nodules were marked on the chest CXR (PA view) using an in-house software. Reading cases including initial and F/U CXRs were done at one session.

### Evaluation

To evaluate reproducibility, percent positive agreement (PPA)^21,22^ and Chamberlain’s percent positive agreement (CPPA) were used and can be defined as follows.

1 $${\rm{PPA}}=100\times \frac{2a}{2a+b+c}$$

2 $${\rm{CPPA}}=100\times \frac{a}{a+b+c}$$

**Figure 3:**
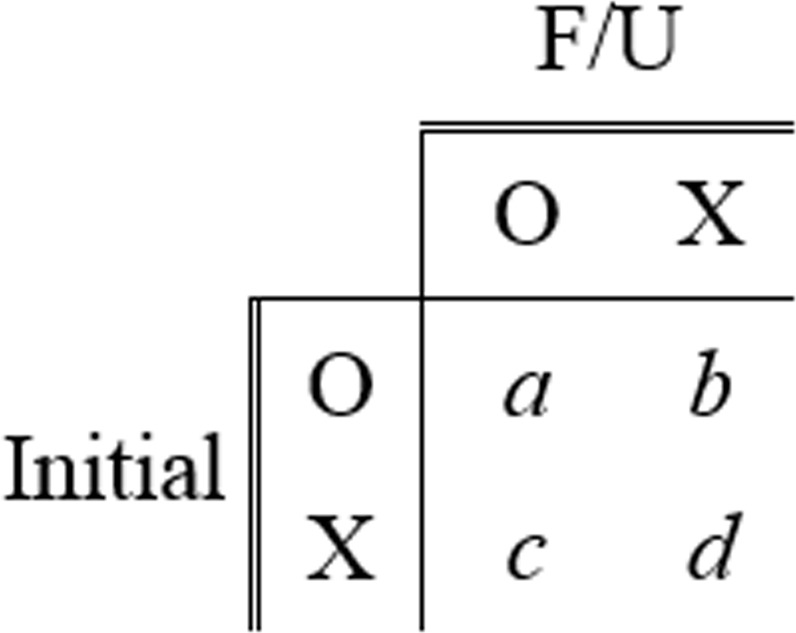
Example of a confusion matrix for reproducibility analysis using initial and F/U CXRs.

Here, *a* is the number of cases in which the same nodule was detected in initial and F/U CXRs and *b* and *c* are the number of cases in which nodules were detected only in initial or F/U CXRs, respectively. Meanwhile, we defined another parameter, *d*, as the number of cases in which a given nodule was not detected in both initial and F/U CXRs; *d* was not used for measurements, such as PPA or CPPA, because our concept is to measure how consistently deep-learning models predicted lesions in patients with diseases that manifest nodules or masses in F/U CXRs. Figure 3 shows an example of a confusion matrix used to measure PPA and CPPA.

## Results

**Figure 4:**
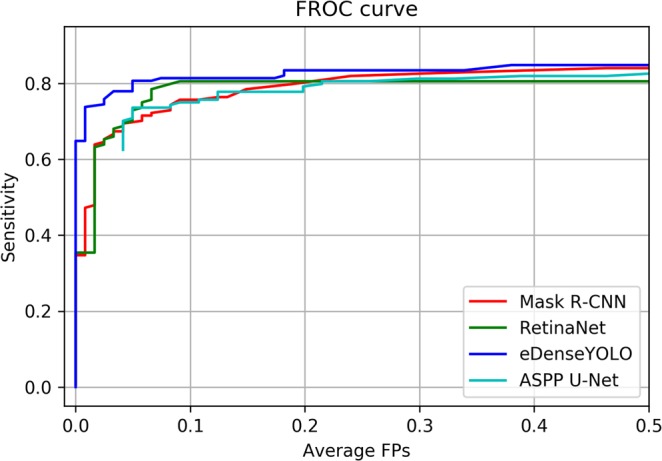
FROC comparison for nodule detection by Mask R-CNN, RetinaNet, eDenseYOLO, and ASPP U-Net.

The reproducibility of the four different chosen CNN algorithms and two chest radiologists (R1 and R2) for nodule detection in CXRs was evaluated. The performance of each CNN-based model is shown in Fig. 4 in terms of FROC curves for the validation set.

**Figure 5:**
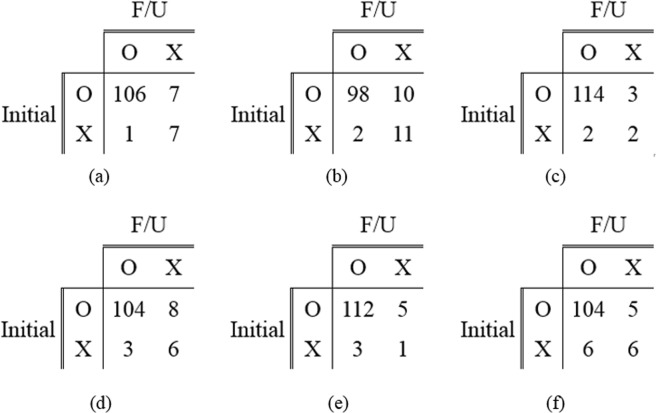
Confusion matrices for measuring reproducibility in initial and F/U CXRs. (**a**) Mask R-CNN, (**b**) RetinaNet, (**c**) eDenseYOLO, (**d**) ASPP U-Net, (**e**) R1, and (**f**) R2.

Five different cut-off thresholds for reproducibility were determined as the number of average false positives (0.1, 0.2, 0.3, 0.4, and 0.5) in FROC curves for evaluating the reproducibility of the validation set. Five PPAs obtained by each model with the five different cut-off thresholds were averaged. In the same manner, CPPAs of each model were averaged. At the cut-off threshold 0.2, the sensitivities 0.80, 0.81, 0.83, and 0.79 were observed for Mask R-CNN, RetinaNet, eDenseYOLO, and ASPP U-Net, respectively. Figure 5 shows the confusion matrices used for measuring reproducibility with initial and follow-up CXRs generated by the four different algorithms and two readers. Table 3 shows the figure of merit (FOM) of jackknife free-response receiver operating curve (JAFROC) and reproducibility of the four different algorithms and two readers in terms of PPA and CPPA. The PPA values were evaluated at 96.52% ± 0.51%, 94.23% ± 0.00%, 97.87% ± 0.08%, 95.04% ± 0.11%, 96.55%, and 94.98% for Mask R-CNN, RetinaNet, eDenseYOLO, ASPP U-Net, R1, and R2, respectively. The CPPA values were calculated to be 93.18% ± 0.81%, 89.09% ± 0.00%, 95.80% ± 0.22%, 90.57% ± 0.19%, 93.33%, and 90.43% for Mask R-CNN, RetinaNet, eDenseYOLO, ASPP U-Net, R1, and R2, respectively. eDenseYOLO exhibited the highest PPA (97.87% ± 0.08%) and CPPA (95.80% ± 0.22%). Similar results were observed in all cases with respect to the relationship between PPA and CPPA.

**Table 3:** Figure of merit (FOM) (95% confidence interval) of jackknife free-response receiver operating curve (JAFROC) and reproducibility comparisons in terms of PPA and CPPA at five-different cutoff values (the number of false positives per CXR was 0.1, 0.2, 0.3, 0.4, and 0.5) of four CNNs based detection algorithms and two readers on nodule and mass case.

Algorithms and readers	FOM (95% CI)	PPA (%)	CPPA (%)
Mask R-CNN	0.87 (0.83–0.91)	96.52 ± 0.51	93.18 ± 0.81
RetinaNet	0.84 (0.78–0.88)*	94.23 ± 0.00	89.09 ± 0.00
eDenseYOLO	**0.89 (0.85–0.93)**	**97.87 ± 0.08**	**95.80 ± 0.22**
ASPP U-Net	0.85 (0.80–0.89)*	95.04 ± 0.11	90.57 ± 0.19
R1		96.55	93.33
R2	—	94.98	90.43

(*p-value < 0.05 between eDenseYOLO and others for FOM).

**Figure 6:**
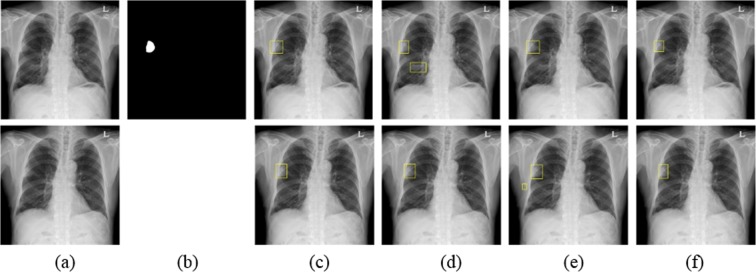
Examples of a mass in the initial and F/U CXRs analyzed by four different algorithms. (**a**) Initial CXR is at the top and F/U CXR is at the bottom. (**b**) Mass mask corresponding to the top of (**a**). The mass is located in the middle lobe of the right lung. (**c**–**f**) Nodule detection in initial and F/U CXRs (top and bottom, respectively) by Mask R-CNN, RetinaNet, eDe nseYOLO, and ASPP U-Net, respectively.

**Figure 7:**
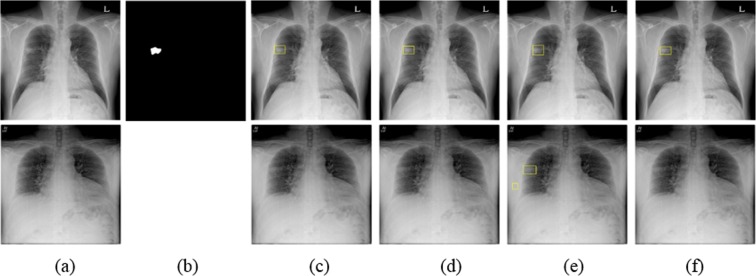
Example of a mass detected by eDenseYOLO in initial and F/U CXRs. (**a**) Initial CXR is shown at the top and F/U CXR is at the bottom. (**b**) Mass mask corresponding to the top of (**a**). The mass is located in the middle lobe of the right lung. (**c**–**f**) Nodule detection in initial and F/U CXRs (top and bottom, respectively) by Mask R-CNN, RetinaNet, eDenseYOLO, and ASPP U-Net, respectively.

**Figure 8:**
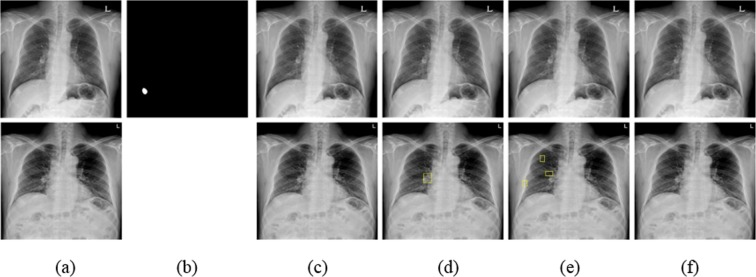
Examples of nodules in initial and F/U CXRs not detected by the four algorithms. (**a**) Initial CXR is shown at the top and F/U CXR is at the bottom. (**b**) Nodule mask corresponding to the top of (**a**). The mass is located in the right upper diaphragm. (**c**–**f**) Mass detection in initial and F/U CXRs (top and bottom, respectively) by Mask R-CNN, RetinaNet, eDenseYOLO, and ASPP U-Net, respectively.

Figures 6–8 show the results obtained using the four different models at a number of false positives of 0.2. Figure 6 shows the CXR of a patient with a mass located in the right upper lobe (3.98 cm as confirmed by CT). The mass was correctly detected in initial and F/U CXRs by the four algorithms and R1 and R2; this case was considered as *a* in the confusion matrix. Figure 7 shows another example of the CXR of a patient with a mass located in the upper lobe (3.36 cm as confirmed by CT). The F/U CXR was blurred compared to the initial CXR. A mass was detected in initial and F/U CXRs by eDenseYOLO, R1, and R2 as shown in Fig. 7(e). Figure 8 shows the CXR of a patient with a mass located in the right lower lobe on the diaphragm (3.41 cm as confirmed by CT). None of the algorithms could detect the mass; it was detected only by R1.

## Discussion

In the past, many researchers measured model performance in terms of sensitivity, specificity, accuracy, AUC, and FROC^10,12–14^. Although these evaluation metrics are important, it is not clear whether these algorithms can perform well in the F/U CXR datasets for short-term periods; it is expected that lung disease patterns observed in the CXRs taken within a short-term period should be detected accurately despite changes in breathing, posture, position, or parameter setting in devices. Therefore, in this study, we undertook to verify the reproducibility of CAD.

The reproducibility of four different algorithms and two chest radiologists in detecting nodules and masses in CXRs was evaluated in terms of PPA and CPPA. In the case of the four algorithms, different PPA and CPPA values were obtained due to a variation in performance metrics, such as sensitivity, at the same standard cut-off threshold. eDenseYOLO showed the highest sensitivity at the cut-off threshold as well as the highest PPA and CPPA. R1 exhibited the second highest PPA and CPPA. Nevertheless, the quality of some F/U CXRs was inferior to that of initial CXRs, which resulted in more false positives or false negatives. Because the F/U CXRs were mainly recorded in emergency situations, they could not be captured in the same conditions as initial CXRs, which resulted in blurry or quite different conditions for CXRs due to motion artifacts in emergency situations and different protocols and machines. In F/U CXRs, eDenseYOLO at a 0.2 threshold (the number of false positives was 0.2) predicted more false negatives (FNs) for small nodules (< 20 mm, FN ratio –  initial: 43%, F/U: 56%) than for large nodules or masses (≥ 20 mm, FN ratio – initial: 12%, F/U: 14%). One of the differences between eDenseYOLO and other algorithms (other than the overall architecture) is the use of a dense block that enables greater information propagation and the inclusion of an ensemble method that makes the model more robust in detecting lesions of various sizes. Thus, we suggest the use of a more efficient encoding block, an ensemble technique, and augmentation methods, such as smoothness, noise, pose rotation, and deformable transform, for training more robust models for variable conditions.

To employ deep-learning-based CAD systems in clinical settings, they must exhibit good performance as imaging biomarkers. Especially in clinical practice, a number of F/U CXRs may be generated for the same patient with no interval changes or minimal changes in nodule and mass, regardless of the inspiration level, position, and radiation dose. Because clinical physicians expect similar reports in F/U CXR evaluation, inconsistent reports are detrimental to a physician’s confidence. Previous conventional CAD systems without deep learning were not used in clinical practice as they generated a number of false positive lesions. However, recent CAD systems using deep-learning methods exhibit very good performance including a high sensitivity with few false positives, which is important for consistent results and clinical applications.

When developing CAD systems, the issue of reproducibility should be considered using multiple CXRs of the same patient in the training and validation sets.

However, our study has some limitations as well. First, we used only a single-center dataset. The trend followed by PPA and CPPA should be checked with more validation sets from multi-center studies. Second, only a simple augmentation method was used to train the model. Other augmentation methods can probably enhance model performance in terms of sensitivity, PPA, and CPPA.

In future, we aim to collect more CXRs and review the current gold standards stored in big data servers. To reduce false positives in reproducibility analyses, we plan to research deep-learning algorithms to use two or more deep-learning networks in CXR CAD and training methods that can perform reproducibly in dataset pairs within short-term periods. In this study, we did not use biopsy information to detect the type of cancer. However, CAD techniques for determining the type of cancer should be developed.

## Conclusions

We suspect that deep-learning-based CAD techniques can help radiologists improve reproducibility in detecting pulmonary nodules. However, we observed in our study that there exist moderate variations in the reproducibility of CAD techniques with different CNN-based detection algorithms, which indicates that reproducibility is an important parameter in evaluating the performance of such techniques in clinical applications. Hence, it is important to train CAD models for reproducibility in paired datasets in medical environments.
